# Microscopy and genomic analysis of *Mycoplasma parvum* strain Indiana

**DOI:** 10.1186/s13567-014-0086-7

**Published:** 2014-08-13

**Authors:** Naíla C do Nascimento, Andrea P dos Santos, Yuefeng Chu, Ana MS Guimaraes, Aubrey N Baird, Ann B Weil, Joanne B Messick

**Affiliations:** Department of Veterinary Pathobiology, Purdue University, 725 Harrison Street, 47907 West Lafayette, IN USA; State Key Laboratory of Veterinary Etiological Biology, Lanzhou Veterinary Research Institute of CAAS, Lanzhou, China; Department of Veterinary Clinical Sciences, Purdue University, 625 Harrison Street, 47907 West Lafayette, IN USA; Present address: Department of Animal Health and Preventive Veterinary Medicine, College of Veterinary Medicine and Animal Science of University of São Paulo, São Paulo, Brazil

## Abstract

*Mycoplasma parvum* [*Eperythrozoon parvum*] is the second hemotrophic mycoplasma (hemoplasma) described in pigs. Unlike *M. suis*, its closest phylogenetic relative*, M. parvum*, is considered a non-pathogenic bacterium in this host species. Natural infection of a domestic, 6-month-old splenectomized pig with *M. parvum* strain Indiana is described herein. Light and scanning electron microscopy of the bacteria were performed in addition to whole genome sequencing, analysis, and comparison to the genome of *M. suis* strain Illinois. Neither clinical signs nor anemia were observed during the infection. Microscopy analyses revealed coccoid to rod- shaped organisms varying from 0.2 to 0.5 μm; they were observed individually or in short chains by both light and electron microscopy, however less than 30% of the red blood cells were infected at peak bacteremia. The single circular chromosome of *M. parvum* was only 564 395 bp, smaller than *M. genitalium*, previously considered the tiniest member of the *Mollicutes*. Its general genomic features were similar to others in this class and species circumscription was verified by phylogenomic analysis. A gene-by-gene comparison between *M. suis* and *M. parvum* revealed all protein coding sequences (CDS) with assigned functions were shared, including metabolic functions, transporters and putative virulence factors. However, the number of CDS in paralogous gene families was remarkably different with about half as many paralogs in *M. parvum.* The differences in paralogous genes may be implicated in the different pathogenic potential of these two species, however variable gene expression may also play a role. Both are areas of ongoing investigation.

**Electronic supplementary material:**

The online version of this article (doi:10.1186/s13567-014-0086-7) contains supplementary material, which is available to authorized users.

## Introduction

Hemotrophic mycoplasmas (class *Mollicutes*), trivially known as hemoplasmas, are small cell-wall-less bacteria with a unique tropism for the red blood cells (RBC) of a variety of vertebrate hosts. The genome of ten hemoplasmas, eight different species and two strains each of *Mycoplasma suis* and *M. haemofelis*, had their genomes sequenced to date; seven of these were completed by our research group [1–10]. Their genomes have a small core of essential genes; however, they rely on the acquisition of most nutrients from their environment to survive. This feature likely plays a key role in the lack of an established in vitro cultivation of these organisms.

*Mycoplasma parvum* [*Eperythrozoon parvum*] was first reported in 1950 as a nonpathogenic bacterium of the pig [11]. Unlike *M. suis*, its closest relative, *M. parvum* often accumulates on a single RBC but only a few RBC are infected. Rod and coccoid forms are common in *M. parvum* infection, ranging in size from 0.2 to 0.5 μm, whereas ring forms are frequent in *M. suis*. The latter form is larger and may be up to 2.5 μm in diameter [11]. Infection by *M. parvum* is usually characterized by the absence of clinical signs even in splenectomized pigs; on the contrary, *M. suis* bacteremia, characterized by fever and icteroanemia, may result in death. In addition, cross-inoculation studies differentiate these two hemoplasmas as separate species as well as from other species of red cell bacteria that infect cattle, sheep, goats, and mice [11,12].

More than 50 years have passed since its first description, and yet less than 10 publications can be found when searching for “*Mycoplasma parvum*” or “*Eperythrozoon parvum*” keywords in scientific databases. The two previous reports documenting *M. parvum* infection in pigs were based solely on the amplification and sequencing of its 16S rRNA or ribonuclease P RNA genes [13,14]. The sequences of these genes were divergent from those of *M. suis*, sharing only 96% and 88% identity, respectively. These molecular studies corroborated previous morphology, pathogenicity, and cross-inoculation studies, suggesting that *M. parvum* and *M. suis* were distinct hemoplasma species. Nevertheless, new molecular tools for species circumscription are likely to provide a better resolution for this complex definition, especially when dealing with these uncultivable bacteria. The average nucleotide identity (ANI) analysis of conserved and shared genes between bacterial strains and supporting calculation of tetranucleotide signature correlation index (TETRA), has recently been proposed as a reliable substitute for DNA-DNA hybridization (DDH) [15]. A value for species delineation based on ANI is currently set at 95%-96%. The TETRA signature is an alignment-free parameter and generally correlates with ANI.

We report herein the course of infection with *M. parvum* in a naturally infected, splenectomized 6-month-old pig, and the complete genome of *M. parvum* strain Indiana. Furthermore, comparative genomic analyses of *M. parvum* with other hemotrophic mycoplasmas were performed, as well as a gene-by-gene comparison between *M. parvum* strain Indiana and *M. suis* strain Illinois to better understand their contrasting pathogenicity.

## Material and methods

### Animal and *Mycoplasma parvum* DNA isolation

A male domestic 6-month-old pig (*Sus scrofa domesticus*, mixed breed) was purchased from the Animal Sciences Research and Education Center-ASREC (West Lafayette, IN, USA). While at the farm, this animal was kept in close contact with other pigs fed a commercial feed containing chlortetracycline (100 grams/ton) during growing and finishing stages (from 7–8 weeks to 6 months old), and vaccinated against *Mycoplasma hyopneumoniae* (RespiSure®/ER Bac Plus®, Pfizer), *Erysipelothrix rhusiopathiae*, and circovirus. The animal was tested for hemotrophic mycoplasma infection [16] on two separate occasions prior to its arrival at the Purdue Animal Housing Facilities (Purdue University, West Lafayette, IN, USA). A conventional PCR (cPCR) developed in our laboratory, which amplifies a larger product (~840 bp) of the 16S rRNA gene of swine hemoplasmas, was also performed [17]. The identity of both qPCR and cPCR products was confirmed by Sanger sequencing. The animal was treated according to the Purdue Animal Care and Use Committee (PACUC) protocol number 1111000223. The pig was fed antibiotic-free commercial feed, as well as water (via a self-controlled nipple waterer) *ad libitum* during the entire study. Eleven days after its arrival, the animal was splenectomized according to the PACUC protocol. The pig was monitored daily (minimum twice a day) for clinical signs (e.g. elevated body temperature, and direct observation of behavior- BAR *status*), and blood was collected into EDTA tubes before and following splenectomy (every 2–8 days depending on clinical signs and blood smear evaluation) for monitoring *M. parvum* infection by qPCR [16].

*M. parvum* strain Indiana was harvested from the blood of the pig at the peak of bacteremia; EDTA-whole blood was centrifuged at 4000 *g* for 10 min and the buffy coat was removed. The remaining red blood cells with *M. parvum* organisms attached were used for the subsequent DNA extraction. Genomic DNA was extracted using Quick-gDNA™ MidiPrep kit according to the manufacturer’s instructions (Zymo Research, Irvine, CA, USA).

### Light and scanning electron microscopy (SEM) of *M. parvum*

Blood smears were prepared from fresh EDTA-blood every 2 days after splenectomy to follow *M. parvum* infection and stained with Giemsa. Photomicrographs were taken using a total magnification of 100 X.

For SEM, aliquots of 1.0 mL of blood infected with *M. parvum* were centrifuged and pellet was resuspended in 1.0 mL of glutaraldehyde 2.5% in 0.1 M sodium cacodylate buffer, pH 7.4, for fixation. Samples were prepared for scanning electron microscopy using standard procedures at Purdue University’s Biological Electron Microscopy Facility, and visualized using FEI Titan Krios microscope.

### Sequencing and assembly of *M. parvum* strain Indiana genome

The whole genome was sequenced from paired-end libraries (TruSeq DNA sample preparation kit, Illumina, San Diego, CA, USA) using Illumina HiSeq 2000 (Illumina, Inc., San Diego, CA, USA) at Purdue University Genomics Core Facility. Average reads of about 100 bases were assembled using ABySS 1.2.7 [18]. After assembly resulting from 1000 × genome coverage of the Illumina reads, a single remaining gap was closed using conventional PCR followed by Sanger sequencing in both directions.

### Annotation and analyses of the complete genome of *M. parvum* strain Indiana

NCBI’s prokaryotic Genomes Annotation Pipeline 2.0 provided the first pass annotation of *M. parvum* genome. The annotation tool Manatee (Institute for Genome Sciences (IGS), School of Medicine, University of Maryland) was used to perform manual annotation of the genome and comparative analysis between the genomes of *M. parvum* and *M. suis* strain Illinois (described below). Genomic data of other mycoplasmas available in the NCBI database (NCBI, Bethesda, MD, USA) were also used for comparative analyses.

Metabolic pathways were predicted based on the KEGG pathway database [19] and the study reported by Yus et al. [20]. BLASTclust tool, by Max-Planck Institute for Developmental Biology [21], was used to assign the paralogous gene families with thresholds of 70% covered length and 30% sequence identity.

### Analyses for species differentiation

JSpecies software was used to calculate the average nucleotide identity (ANI; MUMmer algorithm) and tetranucleotide signature correlation indexes between selected genomes as previously described [15]. The following genome sequences were used in the analyses: *M. parvum* strain Indiana [CP006771.1], *M. suis* strain Illinois [CP002525.1], *M. suis* strain KI3806 [FQ790233.1], *M. haemofelis* strain Ohio2 [CP002808.1], *M. haemofelis* strain Langford 1 [FR773153.2], *M. haemocanis* strain Illinois [NC_016638.1], *M. wenyonii* strain Massachusetts [NC_018149.1], “*Candidatus* M. haemominutum” strain Birmingham 1 [HE613254.1], and “*Candidatus* M. haemolamae” strain Purdue [NC_018219]. The thresholds for species circumscription are 94% and 0.99 for ANIm and tetranucleotide indexes, respectively [15].

### Comparative genomics of *M. parvum* and *M. suis* strain Illinois

Comparative analyses of the whole genome of *M. parvum* and *M. suis* strain Illinois were performed using BLASTp and/or BLASTn of each CDS or gene of one genome against the other and *vice versa*. BLASTp was applied to identify unique CDS of *M. parvum* or *M. suis*; a CDS was considered unique when there were no matching sequences with ≥ 80% coverage and ≥ 40% or ≥ 90% coverage and ≥ 30% identity to the query sequence.

Each unique CDS of *M. parvum* and *M. suis* was analyzed for the identification of the following parameters: subcellular localization and protein sorting signals using PSORTb v3.0.2 [22,23]; signal peptide cleavage sites using SignalP 4.1 [24]; and the presence of lipoproteins using LipoP version 1.0 software [25].

Whole genome synteny (gene order) was compared between *M. parvum* strain Indiana and *M. suis* strain Illinois using SynMap from CoGe [26]. SynMap generates two-dimensional dot-plot synteny maps using a DAGchainer algorithm coupled with BLAST to identify syntenic homologous genes; each dot represents putative homologous genes between any two genomes [27].

## Results

### *Mycoplasma parvum* infection in a splenectomized pig: bacterial loads and clinical signs

**Figure 1:**
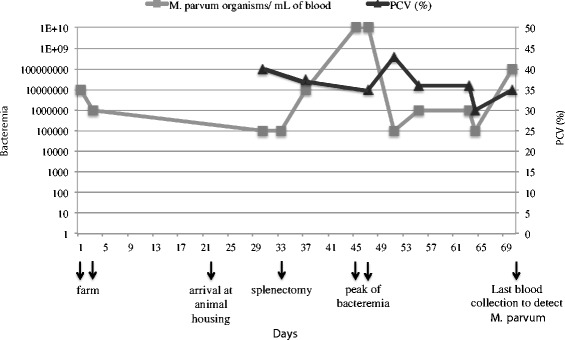
***M. parvum*** **loads and PCV of a naturally infected pig before and after splenectomy.** The loads of *M. parvum* strain Indiana organisms were detected by TaqMan qPCR on blood samples and the (packed cell volume) PCV was determined before and after splenectomy in the naturally infected pig.

A 6-month-old barrow, domestic pig naturally infected with *M. parvum* was used in this study. The dynamics of its *M. parvum* blood load is shown in Figure 1. Briefly, this animal was initially identified as positive using a specific TaqMan qPCR for swine hemoplasmas [16] while still at the farm (ASREC) (days 1 and 3, Figure 1). In addition, a cPCR employed for amplification of ~840 bp fragment of the 16S rRNA gene of swine hemoplasmas was also positive [17]. Sequencing of the qPCR (157 bp) and cPCR (840 bp) products showed 100% and 99% identity, respectively, to the 16S rRNA sequence described for *M. parvum* (GenBank: JX489599.1), in contrast to 97% identity when compared to the 16S rRNA sequence of *M. suis* strain Illinois (NCBI: NR_103930.1). Moreover, the sequencing chromatograms showed single peaks along the entire length of the amplicons indicating the presence of a single sequence. These results confirmed that the animal was infected with *M. parvum* and free of *M. suis*. At day 22, this pig was transferred to the Purdue Animal Housing Facilities, where it remained for 48 days, from April 11^th^ (day 22) to May 29^th^ (day 70) 2013 (Figure 1). The animal was then splenectomized 11 days after its arrival (day 33, Figure 1) according to the Purdue Animal Care and Use Committee protocol (1111000223). Bacterial loads varied from 10^5^ to 10^10^ organisms/mL of blood throughout the course of infection with a peak of bacteremia (10^10^ organisms/mL of blood) occurring 12–14 days after its splenectomy (days 45–47, Figure 1). Organisms were only detected on the peripheral blood smears at peak and for 24 h thereafter. While the pig’s body temperature minimally elevated to 103.7 °F (reference range 101.6-103.6 °F) [28] at the peak, no overt clinical signs of infection were observed. The pig’s hematocrit varied from 30% (only on one occasion, not at the peak of bacteremia; Figure 1) to 43%, but otherwise remained within the reference interval (32-50%) throughout the course of infection [29].

### Light and Scanning Electron Microscopy (SEM) of *M. parvum* organisms

**Figure 2:**
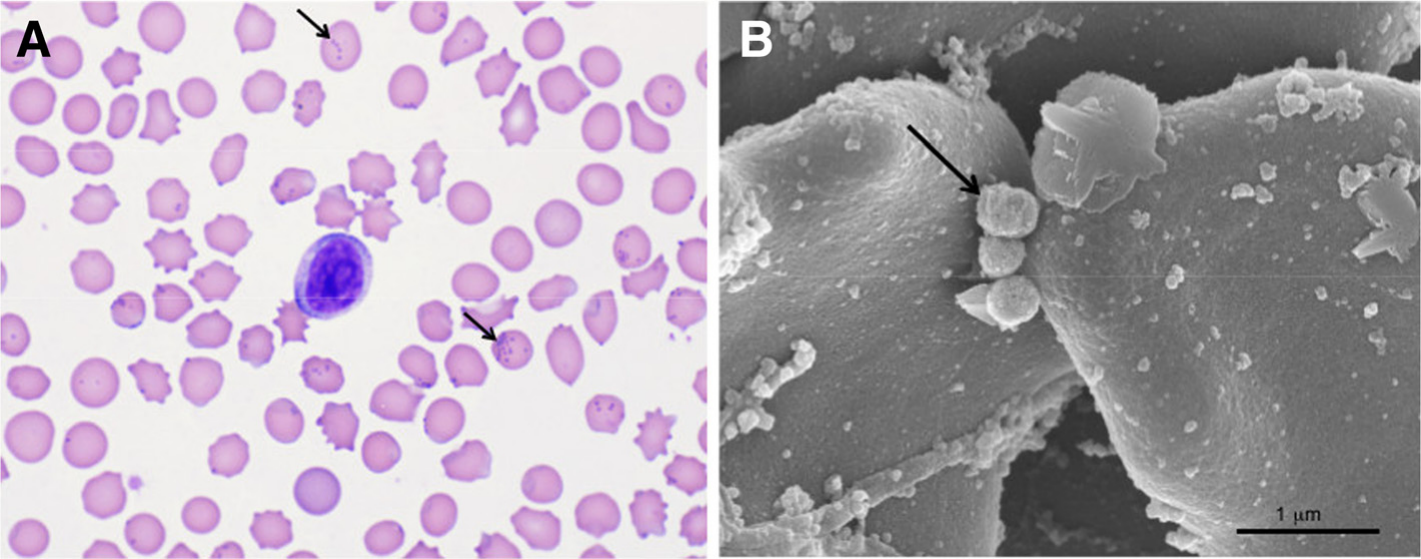
**Light microscopy and SEM of** ***M. parvum*** **strain Indiana organisms. A)** Photomicrograph of a blood smear stained with Giemsa obtained from a pig infected with *Mycoplasma parvum* (black arrows) at the peak of bacteremia (100X magnification). **B)** Scanning electron micrograph (SEM) showing red blood cells (RBC) of the pig with *M. parvum* organisms (black arrow) on the RBC surface. Scale bar, 1.0 μm.

A photomicrograph of the peripheral blood smear obtained at the peak of bacteremia with *M. parvum* (day 45, Figure 1) shows the shape and distribution of these bacteria (Figure 2A). As described previously [11], the organisms are small (approximately 0.2-0.5 μm in diameter), and often infect a few RBC with large numbers of organisms on a single RBC (Figure 2). The SEM shows three *M. parvum* organisms on the surface of a red blood cell; their size varied from 0.2-0.5 μm in diameter (Figure 2B).

### General features of the genome of *M. parvum* strain Indiana

The general features of the genome of *M. parvum* strain Indiana regarding size, G + C content, percentage of coding sequences and paralogous genes were within the range of those described for all hemoplasmas sequenced to date [1,4–8,10] (Table 1). *M. parvum*’s single circular chromosome of 564.395 Kb, the second smallest genome amongst all hemoplasmas, and even smaller than that of *M. genitalium*, which was previously considered the tiniest member of the *Mollicutes* (Table 1). It has a G + C content of 27%, and like other mycoplasmas, it appears to use the opal stop codon (UGA) for tryptophan. The 16S, 23S, and 5S rRNA genes are represented as single copies; however, like that of *Mycoplasma suis*, the 16S rRNA gene is separated from the 5S-23S rRNA in a different operon [1,2]. Thirty-two tRNA were identified covering all amino acids.

**Table 1:** **General features of the genome of** ***Mycoplasma parvum*** **strain Indiana compared to other** ***Mycoplasma*** **species of the pneumoniae group.**

***Mollicutes***												
	**Pneumoniae Group**											
	**Hemothrophic mycoplasmas**								**Mucosal mycoplasmas**			
**Genomic feature**	***M. parvum*** **strain Indiana**	***M. suis*** **strain Illinois**	***M. haemofelis*** **strain Ohio2**	“***Candidatus*** **M. haemominutum**” **strain Birmingham 1**	***M. haemocanis*** **strain Illinois**	***M. wenyonii*** **strain Massachusetts**	“***Candidatus*** **M. haemolamae**” **str. Purdue**	***M. ovis*** **strain Michigan**	***M. pneumoniae***	***M. gallisepticum***	***M. genitalium***	***M. penetrans***
Size (base pairs)	564 395	742 431	1 155 937	513 880	919 992	650 228	756 845	702 511	816 394	1 012 800	580 076	1 358 633
G + C content	27	31.1	38.8	35.5	35.3	33.9	39.3	31.7	40	31	31.7	25.7
Number of genes	616	884	1584	582	1207	687	961	886	733	817	524	1069
Number of Coding sequences (CDS)	581	844	1549	547	1173	652	925	840	689	763	475	1037
CDS with assigned functions	287 (49.4%)	293 (34.7%)	299 (19.3%)	219 (40%)	286 (24.3%)	309 (47.4%)	280 (30.3%)	323 (38.4%)	333 (48.3%)	469 (61.5%)	323 (68%)	585 (56.4%)
Number of rRNA												
16S	1	1	1	1	1	1	1	2	1	2	1	1
23S	1	1	1	1	1	1	1	1	1	2	1	1
5S	1	1	1	1	1	1	1	1	1	3	1	1
Number of tRNA	32	32	31	32	31	32	33	32	37	32	36	29
Number of CDS in paralogous families	141 (24.3%)	361 (42.8%)	1103 (71.2%)	134 (24.5%)	748 (63.8%)	371 (56.9%)	454 (49.1%)	265 (31.5%)	132 (19.1%)	110 (14.4%)	25 (5.2%)	245 (23.6%)

Data was obtained from GenBank database using the following accession numbers: *M. parvum* strain Indiana [CP006771.1], *M. suis* strain Illinois [CP002525.1], *M. haemofelis* strain Ohio2 [CP002808.1], “*Candidatus* M. haemominutum” strain Birmingham 1 [HE613254.1], *M. haemocanis* strain Illinois [NC_016638.1], *M. wenyonii* strain Massachusetts [NC_018149.1], and “*Candidatus* M. haemolamae” strain Purdue [NC_018219], *M. ovis* strain Michigan [CP006935], *M. pneumoniae* [U00089], *M. gallisepticum* [AE015450], *M. genitalium* [L43967], and *M. penetrans* [BA000026]. Paralogous gene families were assigned using BLASTclust, with 70% coverage and 30% sequence identity thresholds.

A total of 581 protein-coding sequences (CDS) were predicted and putative functions were assigned and manually verified using the Manatee annotation pipeline: 287 CDS have putative functional identities, representing almost 50% of the CDS, while the other 50% were represented by hypothetical proteins. Further, 24.3% of the genome is dedicated to duplicated genes organized in paralogous families, mostly composed of hypothetical CDS (Table 1).

### Hemoplasmas and their phylogenomic relationship based on ANI and Tetranucleotide signature indexes

A phylogenomic comparison based on average nucleotide identity (ANI) and tetranucleotide signature correlation indexes (tetra) was performed amongst selected hemoplasmas with sequenced genomes (Table 2). *M. parvum* had an ANI varying from 83.6% to 89.5% when compared to other hemoplasma genomes, including *M. suis*. The tetra correlation indexes of *M. parvum* with other genomes had a range of 0.47 to 0.88. Both ANI and tetra correlation indexes were below the cutoff values of 94% and 0.99, respectively, for species circumscription.

**Table 2:** Average nucleotide identity* (ANI) and tetranucleotide signature (Tetra) correlation indexes of selected hemotrophic mycoplasmas.

	***M. suis*** **str. Illinois**		***M. suis*** **str. KI3806**		***M. haemofelis*** **str. Ohio2**		***M. haemofelis*** **str. Langford**		***M. haemocanis*** **str. Illinois**		***M. wenyonii*** **str. Massachusetts**		***“Candidatus*** **M. haemominutum” str. Birmingham 1**		***“Candidatus*** **M. haemolamae” str. Purdue**	
	**ANI**	**Tetra**	**ANI**	**Tetra**	**ANI**	**Tetra**	**ANI**	**Tetra**	**ANI**	**Tetra**	**ANI**	**Tetra**	**ANI**	**Tetra**	**ANI**	**Tetra**
***M. parvum*** **str. Indiana**	83.94	0.87987	83.65	0.88079	84.66	0.47545	84.3	0.47625	84.04	0.57312	87.33	0.75006	88.45	0.59954	89.48	0.59873
***M. suis*** **str. Illinois**			97.74	0.997	85.41	0.365	84.83	0.366	85.59	0.452	85.24	0.75777	89.48	0.54917	89.34	0.54636
***M. suis*** **str. KI3806**					85.3	0.372	87.74	0.372	85.5	0.453	85.5	0.7632	89.57	0.56269	88.6	0.55492
***M. haemofelis*** **str. Ohio2**							97.3	0.999	85.11	0.959	85.75	0.45892	84.84	0.4172	85.73	0.60193
***M. haemofelis*** **str. Langford**									85.21	0.962	85.84	0.45953	84.99	0.41409	85.74	0.59729
***M. haemocanis*** **str. Illinois**											85.71	0.51169	85.58	0.45207	86.32	0.62344
***M. wenyonii*** **str. Massachusetts**													90.25	0.52911	88.21	0.62571
***“Candidatus*** **M. haemominutum” str. Birmingham 1**															89.83	0.63665

The genomes used for the analyses are available in GenBank under the following accession numbers: *M. parvum* strain Indiana [CP006771.1], *M. suis* strain Illinois [CP002525.1], *M. suis* strain KI3806 [FQ790233.1], *M. haemofelis* strain Ohio2 [CP002808.1], *M. haemofelis* strain Langford [FR773153.2], *M. haemocanis* strain Illinois [NC_016638.1], *M. wenyonii* strain Massachusetts [NC_018149.1], “*Candidatus* M. haemominutum” strain Birmingham 1 [HE613254.1], and “*Candidatus* M. haemolamae” strain Purdue [NC_018219].

*ANI was calculated using MUMmer algorithm in JSpecies software.

Comparing all these species of hemoplasmas, ANI and tetra correlation indexes were between 83.6%-90.25% and 0.36-0.96, respectively, correctly separating these organisms as different species of mycoplasmas. On the contrary, strains of the same species (*M. suis* Illinois and KI3806; *M. haemofelis* Ohio2 and Langford1) showed ANI and tetranucleotide correlation indexes above the proposed thresholds for species definition, as expected.

### *M. parvum versus M. suis*: similarities and differences at the genomic level

**Figure 3:**
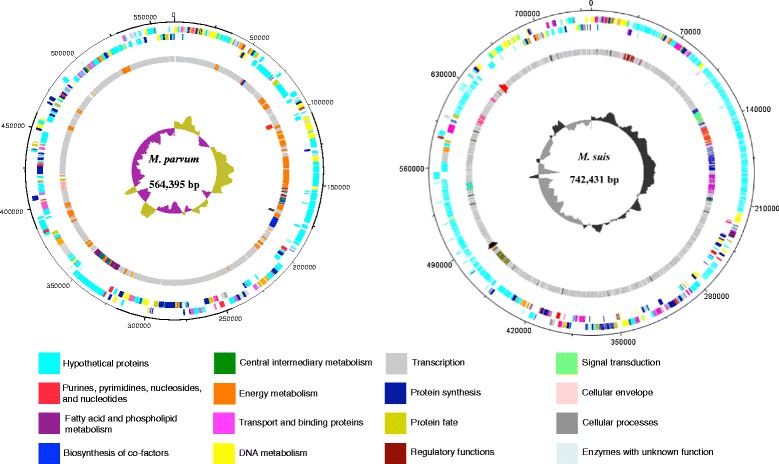
**Circular representation of the genomes of** ***M. parvum*** **strain Indiana and** ***M. suis*** **strain Illinois showing the organization of the coding sequences (CDS).** The dnaA gene is at position zero in both genome plots. Outer to inner circles: circle 1: predicted CDS on the positive strand; circle 2: predicted CDS on the negative strand. Each CDS is classified by TIGR role category according to the color designation in the legend below the plots; circle 3: CDS in the largest paralogous gene families with each family represented by a different color in each genome, non-paralogous CDS are light grey. Paralogous families of *M. parvum* with less than 5 CDS are represented in orange. Black and red marks represent the 16S rRNA gene and the 23S/5S rRNA gene operon, respectively. Circle 4: GC skew. The diagrams were generated using Artemis 12.0 - DNAPlotter version 1.4, Sanger Institute. (*M. suis* plot was extracted from Guimaraes AM, Santos AP, SanMiguel P, Walter T, Timenetsky J, Messick JB: Complete genome sequence of *Mycoplasma suis* and insights into its biology and adaption to an erythrocyte niche. *PLoS One* 2011, 6:e19574 [1], with permission from the copyright holder).

A complete comparison at the genomic level was performed between the genomes of *M. parvum* strain Indiana and *M. suis* strain Illinois (Figure 3). The genome of *M. suis* is 178 Kb bigger than that of *M. parvum*. SynMap analysis (Additional file 1) indicated a lack of overall gene synteny between *M. suis* and *M. parvum*. Although some conserved blocks of genes, mostly corresponding to operons (e.g. PTS system), were observed, they do not necessarily occur at the same relative position of the genomes. A more detailed, manual analysis shows that the 16S rRNA gene and 5S-23S rRNA operon of *M. parvum* are located in the first half of its genome (dnaA at position zero), while *M. suis* copies are in the second half of its genome. Moreover, most of the CDS classified by TIGR role category are organized in a different fashion when comparing both genomes (Figure 3). Despite these rearrangements, both genomes share all CDS with known assigned metabolic or other functions, such as transporters and putative virulence factors; this represents 49.4% and 34.7% of the *M. parvum* and *M. suis* genomes, respectively. The remainder of both genomes consisted in hypothetical CDS, most of which are dedicated to paralogous gene families: *M. parvum* has 141 CDS (24.3%) distributed into 38 paralogous gene families, while *M. suis* has 361 CDS (42.8%) in 68 paralogous gene families (Table 1). Twenty-three out of the 38 paralog families of *M. parvum* are represented in the genome of *M. suis* (with a variable number of members per family between the genomes). In contrast, *M. suis* has 40 out of 68 families that are found exclusively in its genome. This difference in paralogous genes is represented by 188 exclusive CDS in the genome of *M. suis* and 64 CDS unique of *M. parvum*. Considering the hypothetical CDS that are not in paralogous families, both genomes share 74 of these, whereas 63 CDS are found only in *M. parvum* and 153 CDS only in *M. suis*. In total, *M. parvum* has a set of 127 unique CDS, while *M. suis* has 341 CDS found exclusively in its genome.

The set of unique CDS of *M. parvum* and *M. suis* were analyzed for subcellular localization and protein sorting signals [22,23], and the presence of signal peptide cleavage sites [24]. Moreover, the presence of lipoproteins was predicted [25] (Table 3). The majority (54.0-66.5%) of the unique CDS either in paralogous families or not from both *M. parvum* and *M. suis* have an unknown subcellular localization. The remaining CDS are distributed into cytoplasmic membrane, cytoplasmic, and extracellular localization. The unique CDS not in paralogous families from both bacteria are divided mainly into cytoplasmic membrane and cytoplasmic localization, with a smaller number of CDS having a predicted extracellular localization. In contrast, the unique CDS in paralogous families are mostly located in the cytosol, and just a few CDS have cytoplasmic membrane or extracellular localization. The most frequent protein sorting signal amongst all unique CDS is the presence of 1 internal helix, followed by a signal peptide. Analyses of the CDS in paralogous families (details in the Additional file 2) show that a higher number of CDS of *M. suis* (16.5% by PSORTb, and 4.25% by SignalP) have signal peptides compared to *M. parvum* (1.6% bt PSORTb, and 0% by SignalP). Only three lipoproteins (SpII) were identified using LipoP: one unique CDS not paralog of *M. parvum*, one unique CDS not paralog of *M. suis*, and one unique CDS paralog of *M. suis*.

**Table 3:** **Unique CDS of** ***M. parvum*** **strain Indiana and** ***M. suis*** **strain Illinois: subcellular localization and protein sorting signals (PSORTb v3.0.2), presence of signal peptide cleavage sites (SignalP), and presence of lipoproteins (LipoP).**

**Software & parameters**	***M. parvum*** **- unique CDS not in paralogous families**	***M. parvum*** **- unique CDS in paralogous families**	***M. suis*** **- unique CDS not in paralogous families**	***M. suis*** **- unique CDS in paralogous families**
**PSORTb**				
Subcellular localization:				
Unknown	34 (54.0%)	37 (57.8%)	83 (54.2%)	125 (66.5%)
Cytoplasmic Membrane	13 (20.6%)	3 (4.7%)	31 (20.3%)	12 (6.4%)
Cytoplasmic	11 (17.5%)	23 (35.9%)	29 (18.97%)	43 (22.9%)
Extracellular	5 (7.9%)	1 (1.6%)	10 (6.53%)	8 (4.2%)
Features:				
1 internal helix found	30 (47.6%)	20 (31.25%)	77 (50.3%)	153 (81.4%)
2 internal helices found	0	0	7 (4.6%)	0
3 internal helices found	0	0	2 (1.3%)	0
Signal peptide detected	3 (4.8%)	1 (1.6%)	18 (11.8%)	31 (16.5%)
None	30 (47.6%)	43 (67.15%)	49 (32%)	4 (2.1%)
**SignalP**				
YES	3 (4.8%)	0	11 (7.2%)	8 (4.2%)
NO	60 (95.2%)	64 (100%)	142 (92.8%)	180 (95.8%)
**LipoP**				
SpI	8 (12.7%)	9 (14.0%)	36 (23.5%)	65 (34.6%)
SpII	0	1 (1.6%)	1 (0.65%)	1 (0.53%)
TMH	2 (3.2%)	3 (4.7%)	18 (11.8%)	29 (15.4%)
None	53 (84.1%)	51 (79.7%)	98 (64.05%)	93 (49.47%)
**Total**	**63**	**64**	**153**	**188**

PSORTb: results were obtained using the output for Gram-negative bacteria without outer membrane.

SignalP: YES: signal peptide present, NO: signal peptide absent.

LipoP: SpI: signal peptide (signal peptidase I), SpII: lipoprotein signal peptide (signal peptidase II), TMH: n-terminal transmembrane helix. Note from the software: TMH is generally not a very reliable prediction and should be tested. This part of the model is mainly there to avoid transmembrane helices being falsely predicted as signal peptide.

## Discussion

The course of infection with *M. parvum* strain Indiana in the single splenectomized pig evaluated was distinguished from what is commonly seen in *M. suis* infection by the absence of clinical signs, even at the peak of bacteremia. While the bacteremia was apparently fleeting with organisms detected on peripheral blood smears for only a few days after the peak, its persistence at low levels were shown by qPCR. However, these observations are limited to the strain of *M. parvum* described herein and to the animal selected for this study. Further studies should be conducted to evaluate the actual virulence of this isolate, including possible variation among strains and individual pigs. Since the prevalence of *M. parvum* by age is unknown, it is not possible to speculate when this animal got infected with this organism. It is likely, however, that an immune response had developed, which effectively controlled the infection, reducing the bacterial loads. Another possibility is that the use of subtherapeutic doses of antibiotics in the feed at the farm may have controlled the infection. However, the bacteria were not completely eliminated and the animal developed a chronic infection, as is often observed with other hemoplasma species [30]. In contrast, the fever and bacteremia in a splenectomized pig that is infected with *M. suis* is unrelenting and without antibiotics the animal may die [31–33], which was not observed after splenectomy in the animal herein. The impact of acute and chronic hemoplasma infection on the immune system of the host has been poorly explored [34], and is an ongoing area of investigation in our laboratory.

In this report, *M. parvum* strain Indiana was described morphologically by light and scanning electron microscopy and its genome was completely sequenced, analyzed and compared to the genome of *M. suis* strain Illinois. A transient bacteremia was demonstrated by light microscopy, whereas SEM confirmed the rod and coccoid morphology, epicellular location, and small size of *M. parvum* (Figure 2B) [11]. Its size, 0.2 to 0.5 μm, was similar to that reported for other hemoplasmas [35], but remarkably smaller than that reported for *M. suis* with ring forms approaching 1.0 to 2.5 μm in diameter [11,36–38]. The level of bacteremia for *M. parvum* at its peak as shown by qPCR was considerably less (one log) than that previously reported for *M. suis* [16].

*M. parvum* has the smallest single, circular chromosome of all the *Mycoplasma* genomes sequenced to date. The characteristics of the *M. parvum* genome, including its small size, low G + C content, use of UGA codon to encode tryptophan, and number of tRNA and rRNA were in agreement with those reported for the genomes of other hemoplasmas and are typical of mycoplasmas [1,4,6]. In addition, the percentage of CDS dedicated to paralogous genes (24.3%) was similar to that reported for other hemoplasmas [1–8,10]. Our group and others have hypothesized that archived sequences of these genes distributed throughout the chromosome play a role in the ability of mycoplasma organisms to persist despite an active host immune response [39].

Phylogenetic studies of *M. parvum* and *M. suis,* based on sequence analyses of the 16S rRNA and RNase P genes, suggest that these bacteria are closely related [13,14]. Some authors have reported that *M. parvum* is an immature developmental stage of *M. suis* that is present concurrently with the mature pathogen [33,37]. A phylogenomic approach based on ANI and tetranucleotide signatures that uses whole genome sequence information to compare different organisms provides a better resolution than single gene sequence approaches. It is comparable to the method considered as the gold standard for prokaryotic species definition, the DDH [15]. ANI and tetranucleotide results in this study were below the cut-off values for species definition separating all the organisms included in the analyses as distinct species of hemotrophic mycoplasmas. *M. parvum* is, indeed, a distinct species infecting the pig. Further, strains of the same species were clearly distinguished by ANI and tetra scores above the proposed threshold.

*M. parvum* and *M. suis*, share the same host, the pig; the same environment, the blood; and are phylogenetically, the closest related relatives of one another [13,14]. However, the interaction of these bacteria with their host is completely distinct. *M. suis* may cause life threatening hemolytic anemia during acute infection [30,40], while the strain of *M. parvum* shown in this study, and in agreement with previous reports about this organism [11], caused no clinical signs even at the peak of bacteremia in a young, splenectomized pig. At the genomic level, these bacteria are remarkably similar: they share all CDS with known functions. Their genomic synteny, however, is not well conserved. This loss of conservation in the gene order is expected, as different species of hemoplasmas rarely share large syntenic regions, with the exception of *M. haemofelis* and *M. haemocanis* [41]. Nevertheless, the genome of *M. parvum* has orthologous for all the CDS with metabolic functions identified in the genome of *M. suis*, implying that their metabolic pathways work very similarly and they have the same requirements for many nutrients to be acquired from the blood environment [1]. The putative virulence factors described for the *M. suis* genome are also present in *M. parvum*, which raises the question about what is different between these hemoplasmas that reflects in their contrasting pathogenicity. A gene-by-gene comparison between these organisms revealed that their only difference at the genomic level relies in the hypothetical CDS, mostly dedicated to paralogous gene families. *M. suis* has 40 paralogous families that are exclusive to its genome, while *M. parvum* has 15 families that are not found in *M. suis* (Additional file 2). In addition, the largest paralogous family (37 CDS) of *M. suis* is unique. Interestingly, the set of unique paralogous CDS of *M. suis* have a higher percentage of signal peptides detected compared to *M. parvum* CDS; this might indicate that these CDS are secreted or inserted into the membrane and therefore they could play an important role in the pathogenicity of *M. suis* as reported for other bacteria [42,43]. The differences regarding the percentages of signal peptides detected by all three software (PSORTb, SignalP, and LipoP) are due to their distinct prediction algorithms. PSORTb is the only tool amongst the three applied in this study that provides an output for Gram-negative bacteria with no outer membrane or cell wall. It is presumed more accurate for *Mycoplasma* species, which although more phylogenetically similar to Gram-positive organisms, lack a peptidoglycan cell wall.

The maintenance of paralogous gene families in their genomes appears to be a high priority among the hemoplasmas. A prominent role for paralogous genes in the development of antigenic variation has been reported for bacterial species of the genus *Anaplasma*, *Ehrlichia*, *Borrelia* and *Mycoplasma* and linked to virulence as well as persistence of these infections [44]. It is tempting to speculate that differences in paralogous families between *M. parvum* and *M. suis* may be implicated in their differing pathogenic potential*.* However*,* this might also be due to variable expression of core genes, especially genes encoding putative virulence factors or to the presence and variable expression of regulatory RNA. The latter has been previously reported for pathogenic versus non-pathogenic bacteria species of *Listeria* [45], while variable expression of virulence genes was observed for different strains of *Escherichia coli* [46], and *Listeria monocytogenes* [47]. An investigation on the differences at the RNA level between *M. parvum* and *M. suis* is warranted, and it is an ongoing project in our laboratory.

### Nucleotide sequence accession number

The genome of *M. parvum* strain Indiana was deposited in GenBank under the accession number [CP006771.1].

## Additional files

Additional file 1:
**Syntenic map between** ***Mycoplasma suis*** **strain Illinois and** ***M. parvum*** **strain Indiana.** Plots were generated using comparative genomics suite CoGe SynMap (“last” analysis). Each dot represents a matching gene pair. Figure shows the syntenic map (organization of the genes) between *Mycoplasma suis* strain Illinois and *M. parvum* strain Indiana. Additional file 2:
**Unique CDS in paralogous families of** ***M. parvum*** **strain Indiana and** ***M. suis*** **strain Illinois: subcellular localization and protein sorting signals (PSORTb v3.0.2), presence of signal peptide cleavage sites (SignalP), and presence of lipoproteins (LipoP).** Table shows the numbers of unique CDS of *Mycoplasma parvum* strain Indiana and *M. suis* strain Illinois organized in paralogous families. Unique CDS were analyzed for: subcellular localization and protein sorting signals according to PSORTb v3.0.2, presence of signal peptide cleavage sites using SignalP 4.1, and presence of lipoproteins using LipoP version 1.0 software.
