# Supplementary figures and images for: Microscopy and genomic analysis of Mycoplasma parvum strain Indiana
Source: Vet Res. 2014 Aug 13;45(1):86. doi: 10.1186/s13567-014-0086-7 (PMC4423628; doi:10.1186/s13567-014-0086-7)

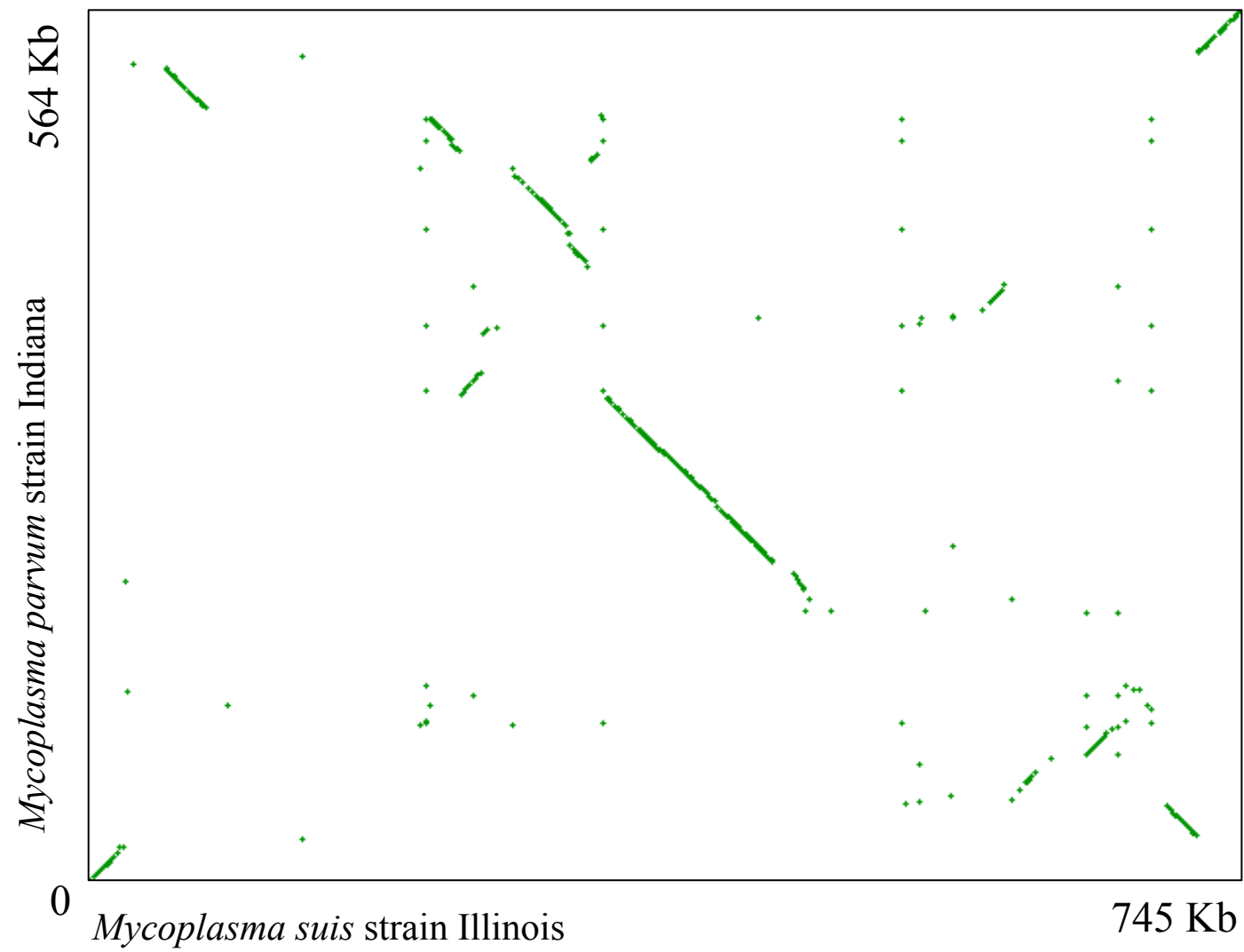

Supplement: Additional file 1: — Syntenic map between Mycoplasma suis strain Illinois and M. parvum strain Indiana. Plots were generated using comparative genomics suite CoGe SynMap (“last” analysis). Each dot represents a matching gene pair. Figure shows the syntenic map (organization of the genes) between Mycoplasma suis strain Illinois and M. parvum strain Indiana. [file 13567_2014_86_MOESM1_ESM.pdf]
