# Supplementary material for: Microscopy and genomic analysis of Mycoplasma parvum strain Indiana
Source: Vet Res. 2014 Aug 13;45(1):86. doi: 10.1186/s13567-014-0086-7 (PMC4423628; doi:10.1186/s13567-014-0086-7)
Supplement: Additional file 2: — Unique CDS in paralogous families of M. parvum strain Indiana and M. suis strain Illinois: subcellular localization and protein sorting signals (PSORTb v3.0.2), presence of signal peptide cleavage sites (SignalP), and presence of lipoproteins (LipoP). Table shows the numbers of unique CDS of Mycoplasma parvum strain Indiana and M. suis strain Illinois organized in paralogous families. Unique CDS were analyzed for: subcellular localization and protein sorting signals according to PSORTb v3.0.2, presence of signal peptide cleavage sites using SignalP 4.1, and presence of lipoproteins using LipoP version 1.0 software. [file 13567_2014_86_MOESM2_ESM.pdf]

| Unique CDSs in paralogous families/Analyses | PSORTb | | | | | | | | | | | | |
|---|---|---|---|---|---|---|---|---|---|---|---|---|---|
| | Subcellular localization | | | | Features | | | | SignalP | | LipoP | | |
| | **Unknown** | **Cytoplasmic Membrane** | **Cytoplasmic** | **Extracellular** | **1 internal helix** | **2 internal helices** | **3 internal helices** | **Signal peptide** | **YES** | **NO** | **SpI** | **SpII** | **TMH** |
| ***M. parvum* strain Indiana** | | | | | | | | | | | | | |
| Family 1 (12 CDSs) | 5 | 0 | 7 | 0 | 1 | 0 | 0 | 0 | 0 | 12 | 3 | 1 | 0 |
| Family 2 (9 CDSs) | 3 | 0 | 6 | 0 | 0 | 0 | 0 | 0 | 0 | 9 | 0 | 0 | 0 |
| Family 4 (6 CDSs) | 4 | 0 | 2 | 0 | 4 | 0 | 0 | 0 | 0 | 6 | 3 | 0 | 2 |
| Family 7 (5 CDSs) | 3 | 0 | 2 | 0 | 0 | 0 | 0 | 0 | 0 | 5 | 0 | 0 | 0 |
| Family 8 (5 CDSs) | 5 | 0 | 0 | 0 | 4 | 0 | 0 | 0 | 0 | 5 | 1 | 0 | 0 |
| Family 11 (4 CDSs) | 3 | 0 | 1 | 0 | 4 | 0 | 0 | 0 | 0 | 4 | 0 | 0 | 0 |
| Family 12 (4 CDSs) | 3 | 0 | 1 | 0 | 0 | 0 | 0 | 0 | 0 | 4 | 0 | 0 | 0 |
| Family 19 (3 CDSs) | 0 | 0 | 3 | 0 | 1 | 0 | 0 | 0 | 0 | 3 | 0 | 0 | 0 |
| Family 22 (3 CDSs) | 1 | 0 | 1 | 1 | 1 | 0 | 0 | 0 | 0 | 3 | 0 | 0 | 0 |
| Family 24 (3 CDSs) | 3 | 0 | 0 | 0 | 2 | 0 | 0 | 0 | 0 | 3 | 0 | 0 | 0 |
| Family 28 (2 CDSs) | 2 | 0 | 0 | 0 | 0 | 0 | 0 | 0 | 0 | 2 | 0 | 0 | 0 |
| Family 30 (2 CDSs) | 1 | 1 | 0 | 0 | 2 | 0 | 0 | 0 | 0 | 2 | 1 | 0 | 1 |
| Family 31 (2 CDSs) | 1 | 1 | 0 | 0 | 1 | 0 | 0 | 0 | 0 | 2 | 0 | 0 | 0 |
| Family 32 (2 CDSs) | 2 | 0 | 0 | 0 | 0 | 0 | 0 | 1 | 0 | 2 | 1 | 0 | 0 |
| Family 35 (2 CDSs) | 1 | 1 | 0 | 0 | 0 | 0 | 0 | 0 | 0 | 2 | 0 | 0 | 0 |
| **Total (64)** | **37** | **3** | **23** | **1** | **20** | **0** | **0** | **1** | **0** | **64** | **9** | **1** | **3** |
| | **Unknown** | **Cytoplasmic Membrane** | **Cytoplasmic** | **Extracellular** | **1 internal helix** | **2 internal helices** | **3 internal helices** | **Signal peptide** | **YES** | **NO** | **SpI** | **SpII** | **TMH** |
| ***M. suis* strain Illinois** | | | | | | | | | | | | | |
| Family 1 (37 CDSs) | 24 | 4 | 9 | 0 | 35 | 0 | 0 | 6 | 1 | 36 | 8 | 0 | 8 |
| Family 7 (11 CDSs) | 5 | 0 | 5 | 1 | 6 | 0 | 0 | 0 | 0 | 11 | 2 | 0 | 0 |
| Family 10 (9 CDSs) | 7 | 0 | 2 | 0 | 5 | 0 | 0 | 3 | 0 | 9 | 5 | 1 | 2 |
| Family 11 (9 CDSs) | 5 | 1 | 3 | 0 | 9 | 0 | 0 | 1 | 1 | 9 | 2 | 0 | 0 |
| Family 14 (8 CDSs) | 8 | 0 | 0 | 0 | 0 | 0 | 0 | 0 | 0 | 8 | 0 | 0 | 0 |
| Family 16 (7 CDSs) | 3 | 0 | 3 | 1 | 6 | 0 | 0 | 0 | 0 | 7 | 2 | 0 | 1 |
| Family 17 (6 CDSs) | 3 | 0 | 3 | 0 | 6 | 0 | 0 | 0 | 0 | 6 | 3 | 0 | 0 |
| Family 18 (6 CDSs) | 6 | 0 | 0 | 0 | 3 | 0 | 0 | 1 | 3 | 3 | 3 | 0 | 0 |
| Family 19 (6 CDSs) | 4 | 2 | 0 | 0 | 5 | 0 | 0 | 0 | 1 | 5 | 4 | 0 | 0 |
| Family 22 (5 CDSs) | 3 | 0 | 2 | 0 | 4 | 0 | 0 | 0 | 0 | 5 | 5 | 0 | 3 |
| Family 23 (5 CDSs) | 3 | 0 | 2 | 0 | 3 | 0 | 0 | 1 | 0 | 5 | 1 | 0 | 0 |
| Family 24 (5 CDSs) | 3 | 0 | 0 | 2 | 5 | 0 | 0 | 2 | 0 | 5 | 3 | 0 | 3 |
| Family 27 (4 CDSs) | 3 | 0 | 0 | 1 | 4 | 0 | 0 | 0 | 0 | 4 | 2 | 0 | 0 |
| Family 28 (4 CDSs) | 4 | 0 | 0 | 0 | 4 | 0 | 0 | 2 | 0 | 4 | 0 | 0 | 0 |
| Family 30 (4 CDSs) | 3 | 1 | 0 | 0 | 2 | 0 | 0 | 1 | 1 | 3 | 2 | 0 | 1 |
| Family 32 (4 CDSs) | 3 | 1 | 0 | 0 | 4 | 0 | 0 | 0 | 0 | 4 | 0 | 0 | 0 |
| Family 33 (4 CDSs) | 3 | 0 | 0 | 1 | 4 | 0 | 0 | 3 | 0 | 4 | 2 | 0 | 1 |
| Family 36 (3 CDSs) | 1 | 1 | 1 | 0 | 3 | 0 | 0 | 0 | 0 | 3 | 0 | 0 | 0 |
| Family 37 (3 CDSs) | 1 | 0 | 2 | 0 | 2 | 0 | 0 | 1 | 1 | 2 | 2 | 0 | 1 |
| Family 38 (3 CDSs) | 0 | 0 | 3 | 0 | 3 | 0 | 0 | 0 | 0 | 3 | 1 | 0 | 1 |
| Family 39 (3 CDSs) | 3 | 0 | 0 | 0 | 3 | 0 | 0 | 0 | 0 | 3 | 0 | 0 | 0 |
| Family 40 (3 CDSs) | 1 | 0 | 2 | 0 | 1 | 0 | 0 | 0 | 0 | 3 | 0 | 0 | 0 |

| | | | | | | | | | | | | | |
|---|---|---|---|---|---|---|---|---|---|---|---|---|---|
| Family 41 (3 CDSs) | 2 | 0 | 0 | 1 | 3 | 0 | 0 | 2 | 0 | 3 | 2 | 0 | 2 |
| Family 42 (3 CDSs) | 1 | 1 | 1 | 0 | 3 | 0 | 0 | 0 | 0 | 3 | 1 | 0 | 0 |
| Family 43 (3 CDSs) | 3 | 0 | 0 | 0 | 3 | 0 | 0 | 0 | 0 | 3 | 2 | 0 | 0 |
| Family 46 (2 CDSs) | 1 | 0 | 0 | 1 | 2 | 0 | 0 | 0 | 0 | 2 | 2 | 0 | 1 |
| Family 48 (2 CDSs) | 1 | 0 | 1 | 0 | 2 | 0 | 0 | 0 | 0 | 2 | 0 | 0 | 0 |
| Family 50 (2 CDSs) | 2 | 0 | 0 | 0 | 2 | 0 | 0 | 0 | 0 | 2 | 0 | 0 | 0 |
| Family 53 (2 CDSs) | 2 | 0 | 0 | 0 | 2 | 0 | 0 | 0 | 0 | 2 | 1 | 0 | 1 |
| Family 54 (2 CDSs) | 1 | 0 | 1 | 0 | 0 | 0 | 0 | 0 | 0 | 2 | 0 | 0 | 0 |
| Family 56 (2 CDSs) | 2 | 0 | 0 | 0 | 2 | 0 | 0 | 2 | 0 | 2 | 1 | 0 | 0 |
| Family 57 (2 CDSs) | 1 | 0 | 1 | 0 | 2 | 0 | 0 | 1 | 0 | 2 | 1 | 0 | 1 |
| Family 58 (2 CDSs) | 2 | 0 | 0 | 0 | 2 | 0 | 0 | 0 | 0 | 2 | 0 | 0 | 0 |
| Family 59 (2 CDSs) | 2 | 0 | 0 | 0 | 1 | 0 | 0 | 0 | 0 | 2 | 1 | 0 | 0 |
| Family 60 (2 CDSs) | 2 | 0 | 0 | 0 | 2 | 0 | 0 | 2 | 0 | 2 | 2 | 0 | 0 |
| Family 61 (2 CDSs) | 2 | 0 | 0 | 0 | 1 | 0 | 0 | 1 | 0 | 2 | 1 | 0 | 0 |
| Family 62 (2 CDSs) | 1 | 0 | 1 | 0 | 2 | 0 | 0 | 0 | 0 | 2 | 2 | 0 | 2 |
| Family 63 (2 CDSs) | 1 | 1 | 0 | 0 | 2 | 0 | 0 | 0 | 0 | 2 | 0 | 0 | 0 |
| Family 65 (2 CDSs) | 2 | 0 | 0 | 0 | 2 | 0 | 0 | 1 | 0 | 2 | 2 | 0 | 1 |
| Family 66 (2 CDSs) | 2 | 0 | 0 | 0 | 2 | 0 | 0 | 1 | 0 | 2 | 0 | 0 | 0 |
| **Total (188)** | **125** | **12** | **43** | **8** | **153** | **0** | **0** | **31** | **8** | **100** | **65** | **1** | **29** |

PSORTb: results were obtained using the output for Gram-negative bacteria without outer membrane.
SignalP: YES: signal peptide present, NO: signal peptide absent.
LipoP: SpI: signal peptide (signal peptidase I), SpII: lipoprotein signal peptide (signal peptidase II), TMH: n-terminal transmembrane helix. **Note from the software: TMH is generally not a very reliable prediction and should be tested. This part of the model is mainly there to avoid transmembrane helices being falsely predicted as signal peptides.**
